# Homemade versus extruded and wet commercial diets for dogs: Cost comparison

**DOI:** 10.1371/journal.pone.0236672

**Published:** 2020-07-24

**Authors:** Thiago Henrique Annibale Vendramini, Vivian Pedrinelli, Henrique Tobaro Macedo, Rafael Vessecchi Amorim Zafalon, Larissa Wünsche Risolia, Mariana Fragoso Rentas, Matheus Vinicius Macegoza, Augusto Hauber Gameiro, Marcio Antonio Brunetto

**Affiliations:** 1 Pet Nutrology Research Center, Nutrition and Production Department, School of Veterinary Medicine and Animal Science, University of Sao Paulo (USP), Pirassununga, Brazil; 2 Veterinary Nutrology Service, Veterinary Teaching Hospital, School of Veterinary Medicine and Animal Science, University of Sao Paulo, Sao Paulo, Brazil; 3 Laboratory of Socioeconomic Analysis and Animal Science, Nutrition and Production Department, School of Veterinary Medicine and Animal Science, University of Sao Paulo (USP), Pirassununga, Brazil; University of Lincoln, UNITED KINGDOM

## Abstract

The present study aimed to verify and compare the costs of homemade diets with extruded and wet commercial diets for dogs in maintenance and consuming therapeutic diets (obesity, congestive heart failure, diabetes, hepatic encephalopathy, chronic kidney disease, and food hypersensitivity), which is important information that impacts in the choice of food by the owners. The maintenance energy requirements (MER) were estimated for adult dogs of different sizes (3 kg, 15 kg, 30 kg, and 50 kg) and the daily amounts of food intake were estimated for each type of food. The costs were calculated per day, per 1000 kcal of metabolizable energy of product and per kg of metabolic weight of the animal. Fourteen complete and balanced homemade diets were formulated, and in each proposed group, two diets with different protein sources were used. Under the conditions of the present study, it was possible to conclude that homemade diets were more expensive than dry maintenance diets and dry therapeutic diets, while commercial wet diets were more expensive in all of the scenarios.

## Introduction

There is a growing interest from dog and cat owners in feeding their pets with homemade diets. Laflamme et al. (2008) [1] conducted a study with pet owners from the USA and Australia and showed that 18% of the animals on the studied population consumed a homemade diet as part or 100% of the daily food intake. In a recent study, an online questionnaire was applied to owners from different regions (Europe, Asia, Africa, and Oceania) and showed that over 60.0% of dogs and cats from 55 countries consumed homemade food as part of their diet, and 12.0% of dogs and 6.0% of cats were fed exclusively with this type of food [2].

Owners' interest regarding homemade foods for dogs and cats may be due to difficulty in understanding labels of processed products, concern with the presence of preservatives and coloring agents, satisfaction in preparing their pet food, and greater palatability [1, 2–5]. Besides those reasons, many dog owners choose homemade diets because they believe it is more affordable when compared to commercial foods, especially concerning therapeutic commercial foods (obesity, congestive heart failure, diabetes mellitus, hepatic encephalopathy, food hypersensitivity, and chronic kidney disease), which are usually more expensive than maintenance commercial foods.

Although it is a topic of great importance and much debated, there are few studies in the literature that verified and compared the costs between complete homemade diets and commercial foods for dogs, which is the objective of the present study.

## Material and methods

The present study was developed between August 2019 and February 2020, in the city of São Paulo, São Paulo, Brazil. A total of fourteen complete and balanced homemade diets were formulated by trained veterinary professionals using the Optimal Formula 2000 software (Optimal Informática, Campinas, Brazil): maintenance (n = 2), obesity (n = 2), congestive heart failure (CHF) (n = 2), diabetes mellitus (n = 2), hepatic encephalopathy (n = 2), food hypersensitivity (n = 2), and chronic kidney disease (n = 2). For each proposed group (maintenance and indicated diseases), two diets with different protein sources were formulated: tilapia and lamb for food hypersensitivity; and chicken or beef for the other diseases. The chemical composition of the ingredients used to formulate the diets was obtained from the Brazilian Table of Food Composition [6] and, when the information was not available, the United States Department of Agriculture National Nutrient Database for Standard Reference was used [7]. All the supplements used in the present study were products recommended for homemade diets for dogs and were selected because they were the only ones in the market to supply nutrients without the need to add other supplements.

The composition of the homemade diets was estimated according to each group of diets. The average chemical composition of macronutrients (crude protein, crude fat, crude fiber, ash, calcium, and phosphorus) of maintenance diets and each group of therapeutic diets was calculated and used to formulate the homemade diets. Specific claims of therapeutic diets were also considered during the formulation process: increased nutrient content for calorie restriction of 50% without nutrient deficiency for obesity; lower sodium for CHF; low carbohydrate assimilation ingredients for diabetes; lower copper, sodium and protein for hepatic encephalopathy; novel carbohydrate and protein for food hypersensitivity; and phosphorus and protein content for chronic kidney disease. Therefore, to achieve the target composition, in some cases, different ingredients were necessary. Table 1 shows the ingredient composition of the homemade diets formulated for dogs for the present study, and Table 2 shows the nutrient composition of the homemade diets.

**Table 1:** Ingredient composition of homemade diets formulated for dogs for the present study, as fed.

Ingredient (%)	Maintenance		Obesity		Congestive heart failure		Diabetes mellitus		Hepatic encephalopathy		Chronic kidney disease		Food hypersensitivity	
	Chicken	Beef	Chicken	Beef	Chicken	Beef	Chicken	Beef	Chicken	Beef	Chicken	Beef	Tilapia	Lamb
White rice	47.9	47.2	39.0	39.6	41.8	41.7	32.4	31.2	68.8	69.1	63.6	65.5	-	-
Potato	-	-	-	-	-	-	-	-	-	-	-	-	65.2	66.8
Chicken breast	33.6	-	30.7	-	34.8	-	26.3	-	-	-	-	-	-	-
Chicken thigh	-	-	-	-	-	-	-	-	9.8	-	18.8	-	-	-
Beef shank	-	34.4	-	-	-	36.1	-	27.0	-	8.4	-	15.5	-	-
Lean beef shank	-	-	-	29.4	-	-	-	-	-	-	-	-	-	-
Tilapia	-	-	-	-	-	-	-	-	-	-	-	-	25.7	-
Lamb	-	-	-	-	-	-	-	-	-	-	-	-	-	26.4
Beef liver	2.4	2.5	5.2	5.5	5.7	5.5	3.7	3.1	1.5	1.5	2.3	2.1	-	-
Carrot	9.9	12.0	7.3	7.6	10.4	10.3	7.1	7.4	7.4	7.6	7.3	7.8	4.1	4.1
Lentil	-	-	-	-	-	-	15.7	15.4	-	-	-	-	-	-
Zucchini	-	-	-	-	-	-	10.5	11.1	-	-	-	-	-	-
Green beans	-	-	7.0	8.1	-	-	-	-	-	-	-	-	-	-
Pumpkin	-	-	6.6	7.0	-	-	-	-	-	-	-	-	-	-
Mozzarella cheese	-	-	-	-	-	-	-	-	5.1	5.2	-	-	-	-
Vitamin and mineral supplement^1^	2.5	2.4	3.0	2.8	-	-	2.5	2.6	-	-	-	-	-	-
Vitamin and mineral supplement with low sodium^2^	-	-	-	-	2.4	2.6	-	-	4.7	4.7	-	-	-	-
Vitamin and mineral supplement without protein additives^3^	-	-	-	-	-	-	-	-	-	-	-	-	1.8	2.0
Vitamin and mineral supplement with low phosphorus^4^	-	-	-	-	-	-	-	-	-	-	5.1	4.5	-	-
Soybean oil	3.8	1.5	0.8	0.1	5.0	2.9	1.9	1.2	2.7	3.5	3.0	4.4	3.2	0.7

^1^Minimum content per kg (as fed): folic acid 18 mg, pantothenic acid 360 mg, biotin 1.8 mg, calcium 112.7 g (minimum) and 124.6 g (maximum), copper 138.3 mg, choline 34 g, iron 1.9 g, phosphorus 50.7 g, iodine 33.6 mg, magnesium 14 g, manganese 120.7 g, niacin 960 mg, potassium 66 g, selenium 8.4 mg, sodium 48 g, taurine 9.4 g, thiamine 134.4 mg, vitamin A 121280 UI, cobalamin 840 mcg, vitamin B2 124.8 mg, vitamin B6 60 mg, vitamin D_3_ 13056 UI, vitamin E 960 UI, vitamin K_3_ 39.2 mg, zinc 1.84 g;

^2^Minimum content per kg (as fed): folic acid 36.1 mg, pantothenic acid 720 mg, biotin 3.6 mg, calcium 112.7 g (minimum) and 124.6 g (maximum), L-carnitine 11.9 g, copper 138.2 mg, choline 34 g, iron 1.9 g, phosphorus 50.7 g, iodine 33.6 mg, magnesium 14 g, manganese 120.7 g, niacin 1920 mg, potassium 66 g, selenium 12.8 mg, sodium 32 g, taurine 11.1 g, thiamine 268.8 mg, vitamin A 121280 UI, cobalamin 840 mcg, vitamin B2 249.6 mg, vitamin B6 120 mg, vitamin D_3_ 13056 UI, vitamin E 2880 UI, vitamin K_3_ 39.2 mg, zinc 1.84 g;

^3^ Minimum content per kg (as fed): folic acid 18 mg, pantothenic acid 360 mg, biotin 1.80 mg, calcium 112.7 g (minimum) and 124.6 g (maximum), copper 138.3 mg, choline 34 g, iron 1.9 g, phosphorus 50.7 g, iodine 33.6 mg, magnesium 14 g, manganese 120.7 g, niacin 960 mg, potassium 66 g, selenium 8.4 mg, sodium 48.0 g, taurine 9.4 g, thiamine 134.4 mg, vitamin A 121280 UI, cobalamin 840 mcg, vitamin B2 124.8 mg, vitamin B6 60 mg, vitamin D_3_ 13056 UI, vitamin E 960 UI, vitamin K_3_ 39.2 mg, zinc 1.84 g;

^4^Minimum content per kg (as fed): folic acid 36.1 mg, pantothenic acid 720 mg, biotin 3.6 mg, calcium 127.7 g (minimum) and 141.2 g (maximum), copper 138.2 mg, choline 34 g, iron 1.9 g, phosphorus 10 g, iodine 33.6 mg, magnesium 8 g, manganese 120.7 g, niacin 1920.9 mg, potassium 66 g, selenium 12.8 mg, sodium 52 g, taurine 9.4 g, thiamine 268.8 mg, vitamin A 121280 UI, cobalamin 840 mcg, vitamin B2 249.6 mg, vitamin B6 120.0 mg, vitamin D_3_ 13056 UI, vitamin E 2880 UI, vitamin K_3_ 39.2 mg, zinc 1.84 g.

**Table 2:** Nutrient composition of homemade diets formulated for dogs for the present study, on dry matter basis, estimated by software^1^.

Nutrient	Maintenance		Obesity		Congestive heart failure		Diabetes mellitus		Hepatic encephalopathy		Chronic kidney disease		Food hypersensitivity	
	Chicken	Beef	Chicken	Beef	Chicken	Beef	Chicken	Beef	Chicken	Beef	Chicken	Beef	Tilapia	Lamb
Crude protein (g)	26.0	26.0	38.0	38.0	36.0	36.0	30.0	30.0	14.5	14.5	16.5	16.5	22.0	22.0
Crude fat (g)	14.0	14.0	8.0	8.0	17.0	17.0	11.5	11.5	14.0	14.0	18.0	18.0	16.0	16.0
Total dietary fiber (g)	3.9	3.9	7.0	7.0	3.0	3.0	7.3	7.1	3.0	3.0	3.0	2.8	4.0	4.0
Ash (g)	9.5	10.1	11.0	11.0	7.8	9.2	8.2	8.4	12.1	12.1	12.6	11.3	9.9	10.1
Calcium (g)	1.0	1.1	1.2	1.0	1.0	1.0	0.8	0.8	1.5	1.5	1.7	1.4	0.9	1.0
Phosphorus (g)	0.7	0.7	0.8	0.8	0.7	0.7	0.7	0.7	0.8	0.8	0.3	0.35	0.7	0.7
Sodium (g)	0.5	0.5	0.6	0.5	0.2	0.2	0.4	0.4	0.2	0.2	0.3	0.3	0.4	0.5
Copper (mg)	1.8	2.1	2.5	2.5	1.9	2.1	1.6	1.6	0.8	0.8	2.0	2.1	1.3	1.4
Metabolizable energy DM^2^ (kcal/g)	4.44	4.41	4.11	3.89	4.21	4.22	4.30	4.25	4.29	4.31	4.43	4.38	4.25	4.27
Metabolizable energy OM^3^ (kcal/g)	1.57	1.51	1.24	1.21	1.63	1.58	1.31	1.27	1.49	1.52	1.66	1.69	1.09	1.07

^1^Optimal Formula 2000, Optimal Informática, Campinas, Brazil;

^2^DM = dry matter;

^3^OM = original matter, as fed.

For all the ingredients used in the homemade diets, the total correction factor (TCF) as suggested by Ornellas [8] was used. The TCF is a constant for each food resulting from the relation between the gross weight (as bought) and its net weight (after being cleaned and prepared for consumption). Thus, the amount of the product considered for pricing was obtained by accurately calculating what should be required of the ingredients in gross weight.

The prices of ingredients for homemade diets were obtained directly at the establishments of three of the largest supermarket chains in the state of São Paulo. Prices were obtained every two weeks between August 2019 and February 2020, to reduce the impact of price seasonality, and an average of the values was made. The prices of the vitamin and mineral supplements were obtained from the respective company (Complet, Biofarm, Jaboticabal, Brazil), which sells them directly to consumers.

The prices of commercial diets were obtained through the official websites of the three largest Brazilian pet store chains. Mean prices were calculated for each ingredient and diet. For both the ingredients of homemade and commercial diets, the largest manufacturers and brands were always selected in the Brazilian market, and the highest volume packages marketed were always adopted. Nutrient content was obtained from labels for the segments of maintenance and therapeutic commercial diet, and average nutrient composition was then used to formulate the homemade diets.

For therapeutic diets, all commercial products (dry and wet) available in Brazil in the period of price research for obesity (8 dry and 1 wet), CHF (4 dry and 1 wet), diabetes (4 dry and 1 wet), hepatic encephalopathy (2 dry and 1 wet), chronic kidney disease (5 dry and 2 wet), and food hypersensitivity (4 dry and 2 wet) were included.

Maintenance foods are divided according to industry segmentation (super premium, premium, and standard) in the Brazilian market, which is not characterized by any official regulatory companies. This segmentation is determined by manufacturers and is based on labeling characteristics and price, and is generally accepted by consumers as a qualitative criterion that guides purchase decisions [9]. In the present study, this classification was used to broaden the cost evaluation. In each segment of maintenance diets, five commercial products of the largest manufacturers and brands in the Brazilian market were included. In addition to dry foods, five complete and balanced wet foods for the maintenance of adult dogs were evaluated. There is no division of wet foods into different commercial segments in Brazil, therefore this type of food was not divided into segments. In addition, the costs of shipping of the supplements and preparation costs of the homemade diets were not considered.

The maintenance energy requirements (MER) of adult dogs of 3 kg, 15 kg, 30 kg, and 50 kg were estimated. The established weights were defined to presuppose animals in various sizes (small, medium, large, and giant). The majority of dogs domiciled in Brazil fall under the category of "little opportunity or stimulus to exercise" [10], so the equation used to calculate the MER was 95 x (kg of body weight)^0.75^ = kcal/day [11]. The only exception was for obesity diets, for which the energy requirement for weight loss (ERWL) was calculated instead of the MER, using the equation 70 x (target weight)^0.75^ = kcal/day, considering target weight as the initial weight minus 20% [12].

Subsequently, the daily and monthly quantities of food intake were estimated based on the respective metabolizable energy of each diet. For homemade diets, the energy used was estimated by the computer program used, and, for commercial diets, the metabolizable energy available on the label was used. Two methods are used by companies to determine the metabolizable energy of commercial pet foods: feeding trials, using the method of total fecal collection (with or without urine collection), according to AAFCO (2019) [13]; or the use of predictive equations [13]. When this information was not described on the label, it was obtained by contacting the customer service department of the company.

The costs were calculated per day for dogs of each determined body weight, and also per 1000 kcal of metabolizable energy. The results were also presented in the form of costs per kg of metabolic weight, a form of analysis that allows the estimation of the monthly costs of the category for any animal. The prices were obtained in Brazilian reais (R$) and converted to U.S. dollars (US$), considering an exchange rate of R$ 3.85 for US$ 1.00.

## Results and discussion

Tables 3 and 4 show the costs of homemade diets and the average costs of commercial products for dogs in maintenance per day and per 1000 kcal of metabolizable energy, respectively.

**Table 3:** Cost per 1000 kcal of metabolizable energy of homemade and commercial dry and wet diets for maintenance dogs.

Homemade diets		Commercial dry diets			Commercial wet diets (n = 5)
Chicken (n = 1)	Beef (n = 1)	Super premium (n = 5)	Premium (n = 5)	Standard (n = 5)	
US$/1000 kcal	US$/1000 kcal	US$/1000 kcal	US$/1000 kcal	US$/1000 kcal	US$/1000 kcal
2.59	3.73	0.94	0.54	0.55	8.83

**Table 4:** Daily cost of homemade and commercial dry and wet diets for maintenance dogs.

Body weight (kg)	Homemade diets		Commercial dry diets			Commercial wet diets (n = 5)
	Chicken (n = 1)	Beef (n = 1)	Super premium (n = 5)	Premium (n = 5)	Standard (n = 5)	
	US$/day	US$/day	US$/day	US$/day	US$/day	US$/day
**3.00**	0.56	0.81	0.20	0.12	0.12	1.92
**15.00**	1.88	2.70	0.55	0.31	0.38	6.39
**30.00**	3.16	4.54	0.96	0.51	0.64	10.75
**50.00**	4.64	6.66	1.42	0.74	0.94	15.77

According to the data obtained in this research, chicken-based homemade diets presented lower cost than beef-based diets, with a difference of approximately 43.0%. According to data from the Center for Advanced Studies in Applied Economics [14], the average value per kg of chicken meat is lower than that of beef and pork since 2004 in Brazil.

Regarding cost per 1000 kcal and by body weight categories, it can be observed that commercial dry foods present lower costs than wet foods and homemade diets in all commercial segments (super premium, premium, and standard). In addition, some commercial dry diets contained nutraceuticals or other functional ingredients such as sources of omega-3 fatty acids, which would increase the costs of homemade diets even further. Wet foods presented higher costs when compared to dry foods and homemade foods.

The higher cost of wet diets for dogs can be justified by some factors. The extrusion process demands a certain amount of starch [15], and because wet foods have a different process, they contain less starch than extruded diets [16]. Therefore, this type of diet contains fewer carbohydrates and consequently, more fat and protein in its composition. Furthermore, the canned diets usually contain more fresh meats than dry diets. This difference in composition makes the product more expensive since fat and protein are some of the most expensive ingredients in pet food formulation. Moreover, wet foods contain at least 60% of moisture (and can exceed 80%), higher than the ≤ 14% expected in dry foods [11], which results in low energy density, increasing the volume of food intake necessary to meet the energy requirement. As these foods are sold in pouches or cans with small amounts when compared to dry food packages, it is necessary to buy more packages to supply food intake, increasing the cost. The difference in packaging material between dry and wet diets can also influence the cost, which is higher in wet foods. In addition, due to the higher amount of moisture in these products, the volume and weight are increased and may increase the cost of transportation.

It is important to note that the costs involved in the cooking process, such as water, energy, and cooking gas, were not considered, as these costs are very difficult to calculate. In addition, the owners who prepare homemade food spend more time shopping and preparing the food, and uses more space for storing ingredients and meals that must be frozen or refrigerated. However, if these costs were added to homemade diets, the final costs would increase, expanding the difference between the other food categories.

**Fig 1:**
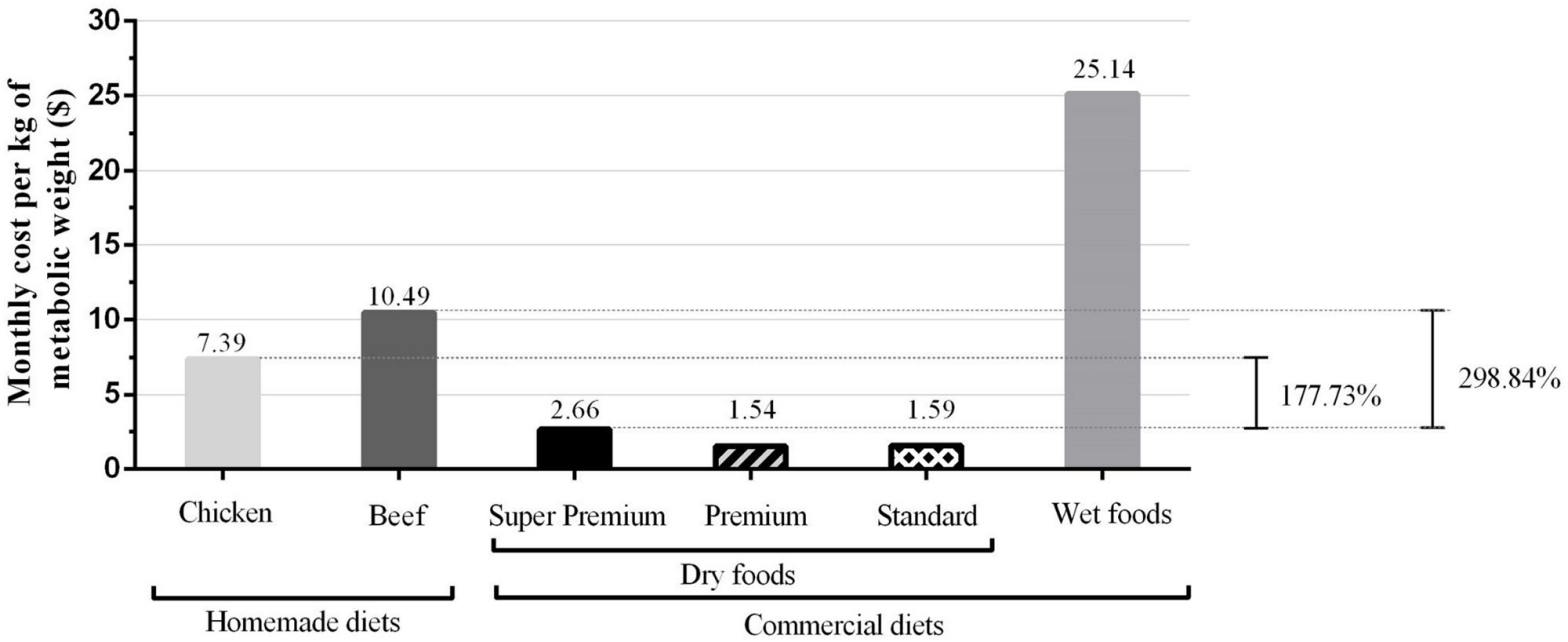
Monthly costs of homemade diets and commercial products for dogs in maintenance per kg of metabolic weight (kg of body weight^0.75^) and relative percentage of costs between super premium and both homemade diets.

When comparing the monthly costs per kg of metabolic weight, the cost of homemade food formulated with chicken is 117.73% higher than super premium commercial food. When formulating with beef, this increase is even higher [on average 298.84% (Fig 1)].

Commercial dry foods of the standard segment, although popularly known and characterized as lower cost [17], when evaluated by cost per 1000 kcal or by intake per category of body weight can be more expensive than premium foods. This difference corresponds, on average, to 1.8% more expensive in the standard segment when compared to the premium segment per 1000 kcal, and up to 27.7% on average, when evaluating intake of a 50 kg dog. This result is justified by the low energy density of standard segment products, due to higher inclusion of vegetable ingredients that contain more fiber and therefore dilute the energy content. A standard commercial diet has an average of 3.4 kcal/kg, while a premium maintenance diet has an average of 3.6 kcal/kg, which represents a difference of 5.8%. Although small, this difference implies that the lower metabolizable energy of the diet corresponds to an increased volume intake, which compensates for the lower cost and tends to increase the owner’s expenditure.

Regarding costs, extruded commercial therapeutic diets are a great alternative for clinical nutrition. In the present study, the dry commercial therapeutic diets had a lower cost than homemade diets, and the wet commercial diets had a much higher cost than both. Tables 5 and 6 show the results obtained for different categories of therapeutic diets considered in the present study.

**Table 5:** Cost per 1000 kcal of metabolizable energy of homemade and commercial therapeutic diets.

Homemade diets		Commercial diets	
Chicken^1^	Beef^2^	Dry	Wet
US$/1000 kcal		US$/1000 kcal	
***Obesity (n = 11)***			
***[2 homemade diets (1 with chicken and 1 with beef) and 9 commercial diets (8 dry and 1 wet)]***			
2.46	3.71	1.85	16.18
***Congestive heart failure (n = 7)***			
***[2 homemade diets (1 with chicken and 1 with beef) and 5 commercial diets (4 dry and 1 wet)]***			
2.46	3.71	1.85	10.92
***Diabetes mellitus (n = 7)***			
***[2 homemade diets (1 with chicken and 1 with beef) and 5 commercial diets (4 dry and 1 wet)]***			
2.77	3.94	2.23	20.91
***Hepatic encephalopathy (n = 5)***			
***[2 homemade diets (1 with chicken and 1 with beef) and 3 commercial diets (2 dry and 1 wet)]***			
3.03	3.21	1.98	10.05
***Chronic kidney disease (n = 9)***			
***[2 homemade diets (1 with chicken and 1 with beef) and 7 commercial diets (5 dry and 2 wet)]***			
3.08	3.19	2.67	8.16
***Food hypersensitivity (n = 8)***			
***[2 homemade diets (1 with tilapia and 1 with lamb) and 6 commercial diets (4 dry and 2 wet)]***			
1.97	5.12	1.89	15.01

^1^For food hypersensitivity the protein source used was tilapia, instead of chicken;

^2^For food hypersensitivity the protein source used was lamb, instead of beef.

**Table 6:** Daily cost of homemade and commercial therapeutic diets.

Body weight (kg)	Homemade diets		Commercial diets	
	Chicken^1^	Beef^2^	Dry	Wet
	US$/day		US$/day	
***Obesity (n = 11)***				
***[2 homemade diets (1 with chicken and 1 with beef) and 9 commercial diets (8 dry and 1 wet)]***				
**3.00**	0.54	0.63	0.36	3.50
**15.00**	1.80	2.11	1.22	11.72
**30.00**	3.03	3.55	2.05	19.71
**50.00**	4.44	5.21	3.00	28.91
***Congestive heart failure (n = 7)***				
***[2 homemade diets (1 with chicken and 1 with beef) and 5 commercial diets (4 dry and 1 wet)]***				
**3.00**	0.53	0.80	0.40	2.36
**15.00**	1.78	2.68	1.34	7.91
**30.00**	2.99	4.51	2.25	13.3
**50.00**	4.39	6.62	3.16	19.5
***Diabetes mellitus (n = 7)***				
***[2 homemade diets (1 with chicken and 1 with beef) and 5 commercial diets (4 dry and 1 wet)]***				
**3.00**	0.60	0.85	0.48	4.53
**15.00**	2.01	2.85	1.61	15.14
**30.00**	3.37	4.79	2.71	25.46
**50.00**	4.95	7.03	3.98	37.34
***Hepatic encephalopathy (n = 5)***				
***[2 homemade diets (1 with chicken and 1 with beef) and 3 commercial diets (2 dry and 1 wet)]***				
**3.00**	0.65	0.70	0.43	2.18
**15.00**	2.19	2.32	1.00	7.28
**30.00**	3.68	3.91	1.56	12.24
**50.00**	5.40	5.74	2.21	17.95
***Chronic kidney disease (n = 9)***				
***[2 homemade diets (1 with chicken and 1 with beef) and 7 commercial diets (5 dry and 2 wet)]***				
**3.00**	0.66	0.69	0.58	1.77
**15.00**	2.23	2.31	1.93	5.91
**30.00**	3.75	3.89	3.25	9.93
**50.00**	5.49	5.71	4.57	14.57
***Food hypersensitivity (n = 8)***				
***[2 homemade diets (1 with tilapia and 1 with lamb) and 6 commercial diets (4 dry and 2 wet)]***				
**3.00**	0.43	1.11	0.41	3.25
**15.00**	1.42	3.71	1.37	10.87
**30.00**	2.39	6.23	2.31	18.28
**50.00**	3.51	9.14	3.11	26.81

^1^For food hypersensitivity the protein source used was tilapia, instead of chicken;

^2^For food hypersensitivity the protein source used was lamb, instead of beef.

**Fig 2:**
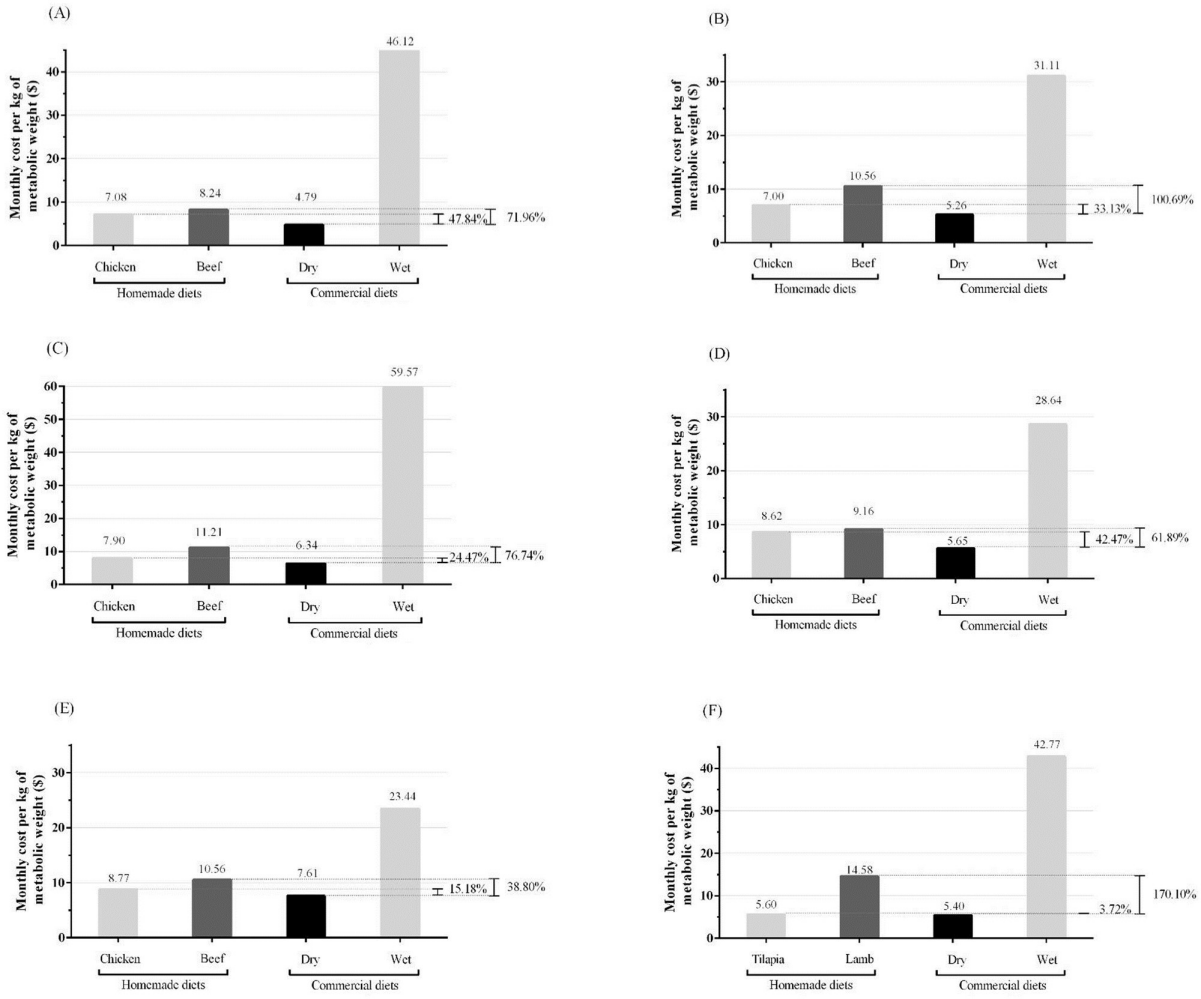
Monthly costs of homemade diets and commercial diets for obesity (A), congestive heart failure (B), diabetes mellitus (C), hepatic encephalopathy (D), chronic kidney disease (E) and food hypersensitivity (F) per kg of metabolic weight (kg of body weight^0.75^) and relative percentage of costs between commercial dry diet and both homemade diets.

For therapeutic diets, in general, the cost of homemade diets containing chicken breast was 15.18% to 52.47% higher than commercial foods, depending on the disease of the animal. When the diet is formulated using beef, this increases to 38.80% up to 100.69% (Fig 2).

The nutritional strategies for each different therapeutic diet are completely different. Diets formulated for weight loss must have characteristics such as low energy density and higher protein content for maintaining lean body mass [12]. Nutrition for dogs with congestive heart failure plays an important role, as it can decrease the rate of disease progression and the development of cachexia, in addition to reducing the pro-inflammatory state and reducing diuretic doses [18], based on sodium restriction and increased protein and fat content.

The objectives of nutrition and management of diabetic dogs are to maintain the energy content, schedule of meals, and nutrient profile, minimizing postprandial fluctuations in blood glucose, especially with the use of slowly assimilated starch [19, 20]. For a patient with hepatic encephalopathy, the dietary protein must be reduced, be highly digestible and have a high biological value to minimize the amount of nitrogen residues available to colon bacteria [21, 22]. Finally, the use of renal therapeutic diets is recommended for patients with stages 2 and up of chronic kidney disease, assessed based on serum creatinine concentrations [23], and these diets are mainly based on the restriction of phosphorus and protein.

When comparing costs of homemade and commercial diets per day and month for dogs with food hypersensitivity, homemade foods were also more expensive than commercial dry foods, for different animal sizes and 1000 kcal. The homemade diet was formulated with tilapia, which is considered as an "unusual" protein source and presented a lower cost than the lamb-based diet.

As with some commercial diets, homemade diets for food hypersensitivity were formulated using unusual protein ingredients (tilapia/lamb and potato as carbohydrate source), which have lower odds of previous exposure by the animals, in relative to commonly found ingredients in commercial diets.

Some commercial diets with hydrolyzed ingredients, which were evaluated in our study (3/4 diets marketed for food hypersensitivity), are recommended for long term treatments [24]. These proteins undergo an enzymatic process that fractionates them to small, low molecular weight peptides. Hydrolysis reduces the molecular weight and intrinsic antigenicity of the food, which reduces the stimuli to the gastrointestinal immune system [25].

The results of all evaluated foods were also presented in the form of monthly costs per kg of metabolic weight to allow the estimation of monthly costs of different categories for any animal. This cost estimate may be relevant and important since professionals can assess the caloric intake for an individual and then use the cost per 1000 kcal value to determine the cost for a specific dog.

The results observed for maintenance dogs in the present study are similar to those of Casna, Shepherd, and Delaney (2017) [26], in a study performed in the United States. These authors found that the cost per 1000 kcal of homemade diets was US$ 3.99, as opposed to US$ 1.20/1000 kcal for dry diets and US$ 5.56/1000 kcal for wet diets. Another study performed by Márquez, Shepherd, and Delaney (2018) [27] evaluated the cost of diets for dogs with chronic kidney disease. In this case, the homemade diet was the least expensive type of diet, followed by dry and wet therapeutic diets (US$ 1.80, US$ 2.18, and US$ 5.71/1000 kcal, respectively). These results differed from those found in the present study, which can be justified by several factors, from the availability and cost of commercial products in different countries, the cost of ingredients, and different ingredients used.

Under the conditions of the present study, it was possible to conclude that homemade diets were more expensive than dry diets for maintenance dogs and more expensive than dry therapeutic diets, and commercial wet diets were more expensive in all of the scenarios. This information is useful for both veterinarians and dog owners, as it is a factor that impacts consumer decision on which product or which type of food to buy and should be approached when making nutritional recommendations.
